# Mapping the Articular Contact Area of the Long Head of the Biceps Tendon on the Humeral Head

**DOI:** 10.1155/2014/814721

**Published:** 2014-08-19

**Authors:** Brent J. Morris, Ian R. Byram, Ray A. Lathrop, Warren R. Dunn, John E. Kuhn

**Affiliations:** ^1^Vanderbilt Sports Medicine, Vanderbilt University Medical Center, Nashville, TN 37232, USA; ^2^Vanderbilt Orthopaedic Institute, 1211 Medical Center Drive, 4200 MCE South Tower, Nashville, TN 37232, USA; ^3^Vanderbilt University School of Engineering, Vanderbilt University, Nashville, TN 37235, USA; ^4^University of Wisconsin Sports Medicine, University of Wisconsin Health, Madison, WI 53792, USA

## Abstract

The purpose of this investigation was to calculate the contact surface area of the long head of the biceps (LHB) in neutral position and abduction. We sought to determine whether the LHB articulates with the humeral head in a consistent pattern comparing articular contact area in neutral position and abduction. Eleven fresh frozen matched cadaveric shoulders were analyzed. The path of the biceps tendon on the articular surface of the humeral head and the total articular surface were digitized using a MicronTracker 2 H3-60 three-dimensional optical tracker. Contact surface area was significantly less in abduction than in neutral position (*P* = 0.002) with a median ratio of 41% (36%, 47.5%). Ratios of contact area in neutral position to full articular surface area were consistent between left and right shoulders (rho = 1, *P* = 0.017) as were ratios of abduction area to full articular surface area (rho = 0.97, *P* = 0.005). The articular contact surface area is significantly greater in neutral position than abduction. The ratios of articular contact surface areas to total humeral articular surface areas have a narrow range and are consistent between left and right shoulders of the same cadaver.

## 1. Introduction

The long head of the biceps (LHB) tendon arises from the posterior superior glenoid labrum and the supraglenoid tubercle and is ensheathed by synovial lining allowing it to be intra-articular yet extrasynovial [1, 2]. It emerges from the glenohumeral joint in the bicipital groove and provides glenohumeral stability acting as a stabilizer and depressor of the humeral head while also elevating the labrum [3–7]. The LHB tendon provides anterior stability of the glenohumeral joint by increasing the resistance to torsional forces in an abducted, externally rotated position in the late-cocking phase [6], while also serving as an important restraint to external rotation in this position [8].

Humeral head chondral lesions thought to originate from excessive contact of the intra-articular portion of the biceps have been described [9, 10]. A “chondral print” has been noted in patients and thought to be an indirect sign of LHB instability [9], and chondromalacia beside the bicipital groove or the “biceps tendon footprint” has also been attributed to maltracking of the LHB in the setting of rotator cuff tears and instability [10]. Pain and stiffness following superior labrum, anterior and posterior lesion (SLAP) repairs may also be associated with a humeral head abrasion under the articular portion of the biceps tendon caused by increased biceps-humeral head contact pressure [11].

Humeral head chondral lesions near the intra-articular portion of the LHB have been noted in patients with SLAP lesions in what was described as a “windshield wiper effect” caused by the LHB [12]. Clinical data was correlated with cadaveric biomechanical data showing humeral head chondral lesions underneath LHB may be due to increased glenohumeral translation and LHB tension in SLAP lesions [13].

Although the LHB tendon has been implicated in the formation of chondral lesions on the humeral head, there is currently no data to identify the articular contact surface area of the LHB. The purpose of this investigation was to calculate the contact surface area of the long head of the biceps on the humeral head in neutral position and abduction. We hypothesized that the LHB would consistently articulate with the humeral head with greater articular contact area in adduction than abduction.

## 2. Material and Methods

Twelve fresh frozen matched cadaveric shoulders of 6 cadaveric specimens with no documented history of rotator cuff disease, arthritis, or prior shoulder girdle injury or surgery were analyzed. One shoulder was excluded after dissection which revealed pathologic disruption of the bicipital sling and medial subluxation of the long head of the biceps. The cadaveric specimens included 4 males and 2 females with average age at time of death of 80 years ranging from 72 to 91 years.

**Figure 1:**
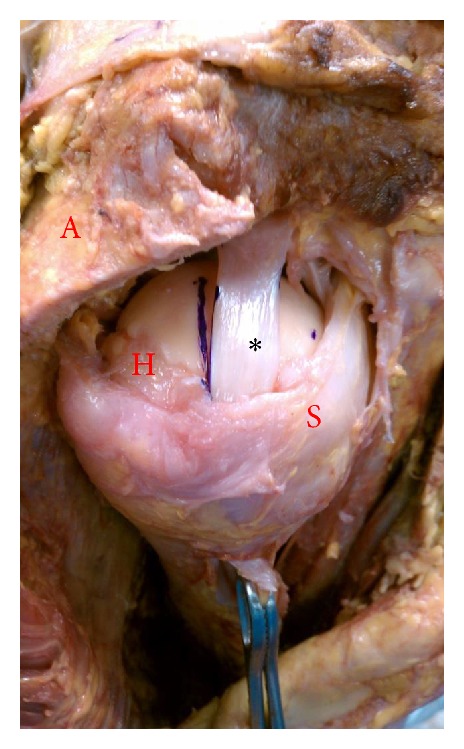
Right shoulder. The biceps tendon (represented as ∗) in neutral position with standard weight maintaining tension on the long head of the biceps tendon and articular contact (A: partially resected acromion for visualization, H: humeral head, and S: subscapularis tendon).

After removal of skin and subcutaneous tissue, each scapula was stabilized with a vice clamp. A 1.5 cm lateral portion of the acromion was removed to improve visualization. A portion of the rotator interval tissue and superior capsule was partially excised in order to visualize the intra-articular portion of the long head of the biceps tendon. Care was taken to preserve the bicipital sling during dissection to prevent iatrogenic subluxation of the LHB. The biceps tendon was exposed distally to the bicipital groove and kept intact. Traction was applied through a 68 gram weight. The weight was applied to ensure that the intact biceps tendon remained taut during range of motion and no subluxation of the tendon was noted (Figure 1).

With the arm in neutral position (0 degrees), the path of the biceps tendon on the articular surface of the humeral head was observed in maximum internal rotation and external rotation. Markings were made with indelible ink on the humeral head along the posterior aspect of the tendon at its most posterior position and then along the anterior aspect of the tendon at its most anterior position. These markings were extended from the exit point of the tendon at the bicipital groove to its most medial contact point on the humeral head. The markings were repeated with the arm abducted 60 degrees in the plane of the scapula, again maximally internally and externally rotating the humerus.

**Figure 2:**
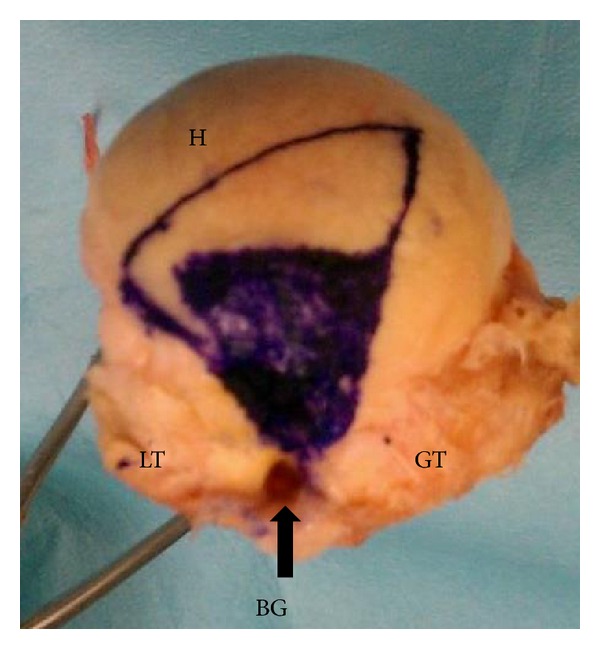
Right humerus. Exposed humeral head with inner trapezoid representing the long head of the biceps contact points in abduction and outer trapezoid representing the long head of the biceps contact points in neutral position (BG: bicipital groove, GT: greater tuberosity, LT: lesser tuberosity, and H: humeral head).

**Figure 3:**
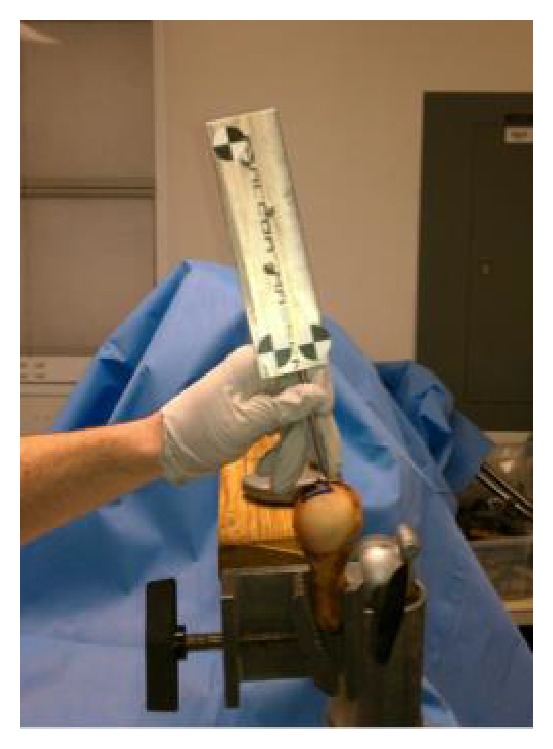
Right humerus. MicronTracker 2 H3-60 three-dimensional optical tracker (Claron Technology Inc., Toronto, Ontario) mapping out the contact surface area.

The remaining soft tissues were removed to expose the humeral head, allowing the markings and entire articular surface to be visualized. The entire articular surface and the two trapezoidal shaped contact areas created in adduction and abduction (Figure 2) were then digitized using a MicronTracker 2 H3-60 three-dimensional optical tracker (Claron Technology Inc., Toronto, Ontario) (Figure 3).

**Figure 4:**
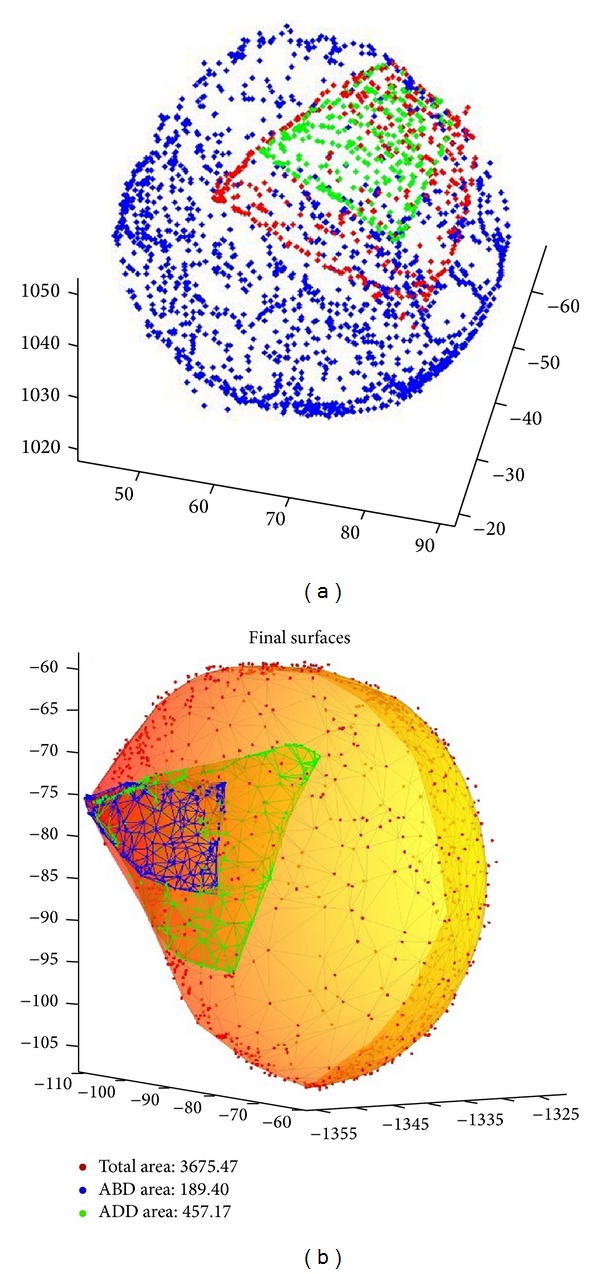
Point clouds representing the three-dimensional surfaces were gathered and smoothed via triangulation using MATLAB software (MathWorks, Natick, Massachusetts), and contact surface areas of the long head of the biceps tendon in abduction, neutral position, and full humeral articular surface were calculated.

Point clouds representing the three-dimensional surfaces were gathered and smoothed via triangulation using MATLAB software (MathWorks, Natick, Massachusetts). Contact surface areas of the biceps tendon and full humeral articular surface were then calculated (Figure 4).

Wilcoxon testing was performed to compare neutral position and abduction contact areas, and Spearman rank correlation was utilized to assess correlation between left and right shoulders. Statistical analysis was performed with free open source R statistical software (http://www.r-project.org/).

## 3. Results

The median articular contact surface area of the biceps tendon (with interquartile range) was 507 (391, 752) mm^2^ in neutral position and 189 (139, 368) mm^2^ in abduction. Median articular surface area of the humerus was 2599 (2090, 3517) mm^2^. Articular contact surface area was significantly less in abduction than neutral position (*P* = 0.002) with a median ratio of 41% (36%, 47.5%). Ratios of articular contact area in neutral position to full articular surface area were consistent between left and right shoulders (rho = 1, *P* = 0.017) as were ratios of abduction articular contact area to complete articular surface area (rho = 0.97, *P* = 0.005).

## 4. Discussion

This cadaveric study was able to quantify the contact surface area of the LHB with the humeral articular surface and qualitatively describe the pathway of the LHB. We were able to demonstrate that the LHB articulates with the humeral head in a consistent pattern, without significant side-to-side variability.

Further work is needed to understand LHB anatomy and pathology to better delineate the association between chondral lesions, the LHB and SLAP lesions. Currently, the mechanism behind this association is not clear. Some have proposed that SLAP lesions lead to instability and subsequent chondral lesions [13], while others believe that an incompetent biceps sling leads to an unstable biceps that can create a humeral chondral lesion [9]. Alternatively, pathologic conditions such as biceps tendonitis may cause resistance to smooth motion in the bicipital groove and cause an increase in articular contact pressures [11]. We believe that the calculation of the contact surface area of the LHB tendon in this investigation provides an important starting point to further demonstrate the role of the LHB tendon in chondral lesions. Our anatomic description may corroborate arthroscopic findings in patients with humeral head articular cartilage lesions [11]. Chondral wear demonstrated in the same trapezoidal pattern originating at the bicipital groove might be attributed to the biceps tendon rather than other sources. Furthermore, we demonstrated that the contact area between the LHB and the humeral head was greater in neutral position than abduction. Patients with a painful rotational arc in neutral position may be reproducing the friction between the LHB and the articular surface, implicating the presence of biceps pathology.

A limitation of this study includes the small sample size. Twelve matched shoulder cadavers were used and one shoulder had to be excluded due to preexisting pathology. A larger sample size could be used to confirm our data; however, we do note consistency side-to-side and among our total cadaver population tested. Furthermore, we sought to measure surface area of LHB articular contact and did not measure contact pressure, which would have strengthened our findings. Another limitation of the study is that a pathologic LHB model was not utilized. We did not assess cadaveric specimens with SLAP lesions or LHB instability, which are suspected to cause chondral lesions on the humeral head. Our goal was to establish a baseline for contact surface area for the normal LHB tendon.

## 5. Conclusions

The articular contact surface area is significantly greater in neutral position than abduction. The ratios of articular contact surface areas to total humeral articular surface areas have a narrow range and are consistent between left and right shoulders of the same cadaver.
